# Synthesis of a Stable Primary-Alkyl-Substituted Selenenyl Iodide and Its Hydrolytic Conversion to the Corresponding Selenenic Acid

**DOI:** 10.3390/molecules201219773

**Published:** 2015-12-02

**Authors:** Shohei Sase, Ryo Kakimoto, Ryutaro Kimura, Kei Goto

**Affiliations:** Department of Chemistry, Graduate School of Science and Engineering, Tokyo Institute of Technology, 2-12-1 Ookayama, Meguro-ku, Tokyo 152-8551, Japan; ssase@chem.titech.ac.jp (S.S.); dolce@m03.itscom.net (R.K.); kimura.r.ad@m.titech.ac.jp (R.K.)

**Keywords:** selenenyl iodide, selenenic acid, hydrolysis, kinetic stabilization, iodothyronine deiodinase

## Abstract

A primary-alkyl-substituted selenenyl iodide was successfully synthesized through oxidative iodination of a selenol with *N-*iodosuccinimide by taking advantage of a cavity-shaped steric protection group. The selenenyl iodide exhibited high thermal stability and remained unchanged upon heating at 100 °C for 3 h in [D_8_]toluene. The selenenyl iodide was reduced to the corresponding selenol by treatment with dithiothreitol. Hydrolysis of the selenenyl iodide under alkaline conditions afforded the corresponding selenenic acid almost quantitatively, corroborating the chemical validity of the recent proposal that hydrolysis of a selenenyl iodide to a selenenic acid is potentially involved in the catalytic mechanism of an iodothyronine deiodinase.

## 1. Introduction

**Scheme 1:**
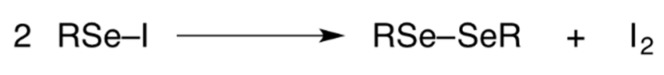
Facile disproportionation of a selenenyl iodide.

**Figure 1:**
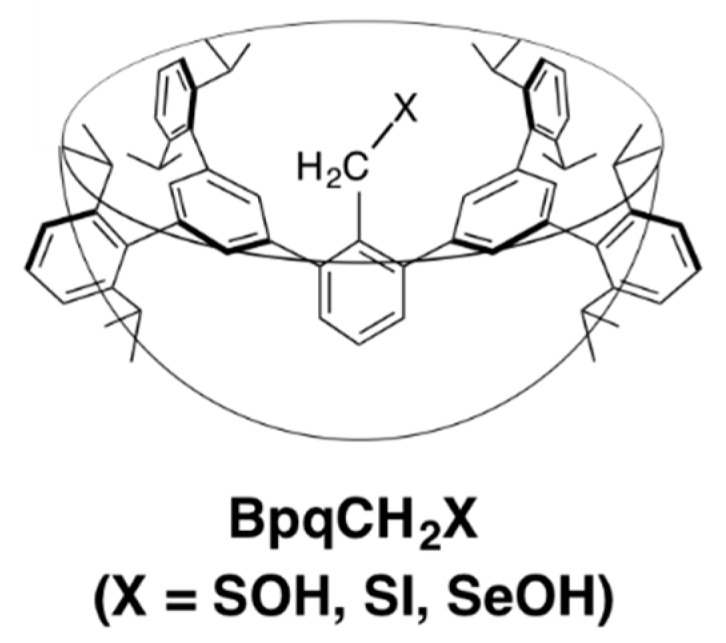
Reactive species stabilized by a cavity-shaped primary-alkyl steric protection group.

Selenenyl iodides (RSeI) have been attracting increasing attention as important intermediates of thyroid hormone deiodination by iodothyronine deiodinases, selenocysteine-dependent enzymes comprising three isoforms (ID-1, ID-2, and ID-3) that catalyze regioselective deiodination of iodothyronines [1,2]. However, model studies on the chemical processes involving the selenenyl iodide intermediates have often been hampered by the notorious instability of the species; selenenyl iodides usually undergo facile disproportionation to the corresponding diselenides and iodine [3,4] (Scheme 1). For the synthesis of stable selenenyl iodides, kinetic stabilization using bulky substituents or thermodynamic stabilization due to intramolecular coordination have been employed [5,6]. We previously succeeded in the isolation of a stable selenenyl iodide bearing a bulky aromatic group and applied it to the demonstration of the chemical transformations included in the catalytic cycle of ID-1 [7,8,9,10]. While all the isolable selenenyl iodides reported so far are aromatic derivatives or tertiary-alkyl derivatives, a primary-alkyl derivative is most relevant as a model compound of naturally occurring selenenyl iodides derived from selenocysteine. It is a common idea, however, that the steric demands of primary-alkyl groups are too small to protect such reactive species, and there has been no example of the synthesis of a primary-alkyl-substituted selenenyl iodide. In the course of our studies on biologically relevant, highly reactive species derived from thiols and selenols, we recently developed a primary-alkyl steric protection group, a BpqCH_2_ group (Figure 1), with a cavity-shaped framework [11]. Despite being a primary-alkyl group, this substituent was found to be effective for the stabilization of reactive species such as a sulfenic acid (X = SOH) [12], a sulfenyl iodide (X = SI) [11], and a selenenic acid (X = SeOH) [13]. Here we report the synthesis of a stable primary-alkyl-substituted selenenyl iodide by utilizing this molecular cavity and its hydrolytic conversion to the corresponding selenenic acid; hydrolysis of a selenenyl iodide to a selenenic acid has been proposed to be potentially involved in the second half-reaction of ID-3 [14], but no chemical evidence has been available for this reaction process.

## 2. Results and Discussion

**Scheme 2:**
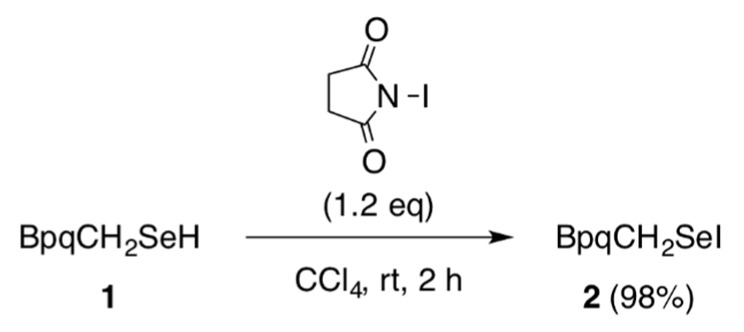
Synthesis of the primary-alkyl-substituted selenenyl iodide **2**.

**Figure 2:**
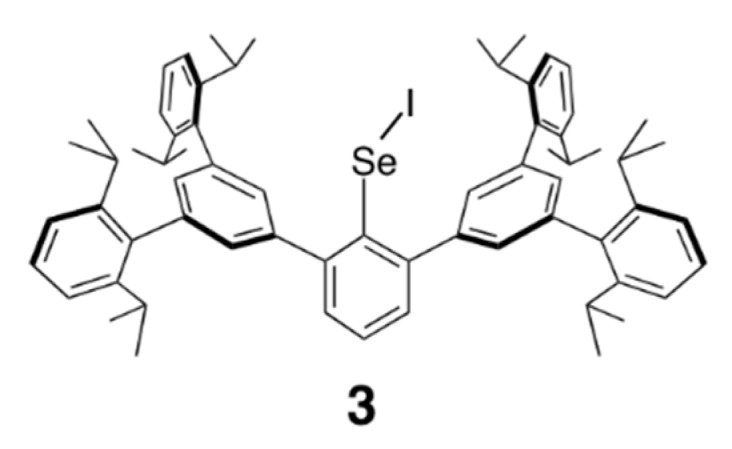
Aryl-substituted selenenyl iodide **3**.

**Scheme 3:**
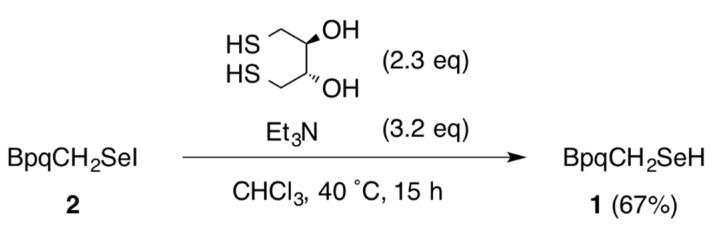
Reduction of selenenyl iodide **2**.

Reaction of selenol **1** [13] bearing the BpqCH_2_ group with 1.2 equivalent of *N-*iodosuccinimide afforded selenenyl iodide **2** in 98% yield (Scheme 2). Selenenyl iodide **2** was isolated as air-stable purple crystals and characterized by NMR (^1^H-, ^13^C-, and ^77^Se-) and UV-vis spectroscopies, mass spectrometry, and elemental analysis. The FD-MS spectrum of **2** showed the molecular ion peak at *m/z* 1090 with a characteristic isotopic pattern of the selenium atom. In the UV-vis spectrum of **2** in CHCl_3_, a weak diagnostic band due to the Se–I functionality was observed at 511 nm. The absorption band of **2** showed a blue shift compared to that of the aryl-substituted selenenyl iodide without the methylene moiety (BpqSeI (**3**), λ_max_ = 553 nm, Figure 2) [7]. In the ^77^Se-NMR spectrum of **2**, a signal was observed at δ 420, which is comparable to that of **3** (δ 465) [7]. The methylene protons adjacent to the Se–I moiety in **2** showed a signal at δ 4.73, which is substantially shifted downfield compared to that of selenol **1** (δ 4.03). A downfield shift was also observed for the α-carbon in ^13^C-NMR spectroscopy (δ 28.2 for **2** and δ 15.3 for **1**). These downfield shifts observed for **2** would be attributable to the electron-withdrawing character of the iodine atom. Seleneyl iodide **2** exhibited high thermal stability. In the solid state, **2** was decomposed at 181–183 °C. In solution, **2** remained unchanged upon heating at 60 °C for 16 h and even at 100 °C for 3 h in [D_8_]toluene. The selenenyl iodide **2** was reduced to the corresponding selenol **1** by dithiothreitol (Scheme 3), as was in the case with the aromatic counterpart, BpqSeI (**3**) [7].

**Scheme 4:**
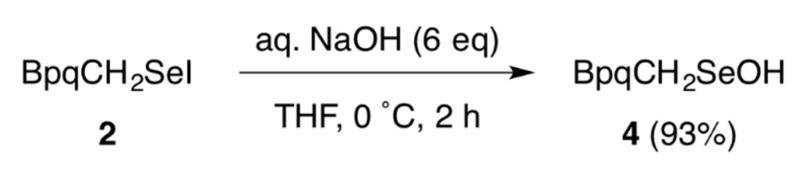
Hydrolysis of **2** to afford selenenic acid **4**.

Sulfenic acids (RSOH) [15,16] and selenenic acids (RSeOH) [17,18] play important roles as reactive intermediates in various biological processes. It is well known that they are formed through the oxidation of cysteine or selenocysteine residues by reactive oxygen species. Meanwhile, in the course of our studies concerning sulfur-containing reactive species, we previously demonstrated that a sulfenic acid can be obtained by alkaline hydrolysis of the corresponding thionitrate (RSNO_2_) or sulfenyl bromide (RSBr) [19]. This corroborates the possible formation of sulfenic acids by similar transformations in biological systems [20,21]. As for the selenium counterpart, it has recently been proposed that related hydrolytic formation of a selenenic acid is potentially involved in the catalytic mechanism of ID-3; Steegborn *et al.* suggested that a selenenyl iodide intermediate generated in the active site of the enzyme after deiodination of an iodothyronine substrate might be hydrolyzed to afford a selenenic acid, which is then reduced to a selenol via a selenenyl sulfide [14]. However, there has been no chemical evidence for the hydrolytic conversion of a selenenyl iodide to a selenenic acid. As a model reaction of this chemical process, the hydrolysis of **2** was examined. The treatment of **2** with an aqueous solution of NaOH at 0 °C for 2 h led to the formation of selenenic acid **4** in 93% yield (Scheme 4). Selenenyl iodides and selenenic acids are intrinsically unstable because of their propensity to undergo facile decomposition via bimolecular pathways. The cavity-shaped BpqCH_2_ group effectively stabilizes both reactive species, enabling the experimental demonstration of this elusive reaction process.

## 3. Experimental Section

Unless otherwise stated, all operations were performed by using high-vacuum and standard Schlenk techniques under an argon atmosphere. Anhydrous tetrahydrofuran (THF) was purchased from Kanto Chemical and passed through a Kayama Oxygen solvent purification system prior to use. CHCl_3_ was washed with conc. H_2_SO_4_ and distilled over CaH_2_. [D_8_]Toluene was distilled from benzophenone ketyl. Other chemicals were purchased from commercial sources and used as received. Preparative thin layer chromatography (PTLC) was performed using Merck silica gel 60 PF_254_ (Darmstadt, Germany). The ^1^H-NMR spectra were recorded on a JEOL ECX-500, a JEOL ECX-400, or a JEOL ECS-400 spectrometer (Tokyo, Japan), and the chemical shifts of ^1^H are referenced to the residual proton signal of CDCl_3_ (δ 7.25). The ^13^C-NMR spectra were recorded on a JEOL ECX-500 or a JEOL ECX-400 spectrometer, and the chemical shifts of ^13^C are referenced to the signal of CDCl_3_ (δ 77.0). All spectra were assigned with the aid of DEPT, COSY, HMQC, and HMBC NMR experiments. The ^77^Se-NMR spectra were recorded on a JEOL ECX-500 or JEOL ECX-400 spectrometer, and the chemical shifts of ^77^Se are referenced to diphenyl diselenide (δ 480) as external standard. UV-vis spectra were recorded on a JASCO V-650 UV-vis spectrometer (Tokyo, Japan). Mass spectra were measured on a JEOL JMS-T100GCv “AccuTOF GCv” spectrometer using a field desorption probe (Tokyo, Japan). Melting points were measured with a Yanaco MP-S3 and are uncorrected (Kyoto, Japan).

### 3.1. Synthesis of BpqCH_2_SeI *(**2**)*

A solution of selenol **1** (15.1 mg, 15.6 µmol) [13] in THF (0.6 mL) in a Schlenk flask with a J-young valve was degassed through three freeze-pump-thaw cycles, and the flask was flushed with argon. To the solution was added a suspension of *N-*iodosuccinimide (4.2 mg, 19 µmol) in CCl_4_ (0.6 mL) at room temperature, and the resulting reaction mixture was stirred for 2 h at room temperature. After evaporation of the solvent, the residue was washed with acetonitrile. Recrystallization from Et_2_O afforded **2** (16.7 mg, 15.3 µmol, 98%) as purple crystals. 

**2**: purple crystals; m.p. 181.4–182.5 °C (dec); ^1^H-NMR (500 MHz, CDCl_3_) δ 1.10 (d, *J* = 6.9 Hz, 24H), 1.16 (d, *J* = 6.9 Hz, 24H), 2.85 (sept, *J* = 6.9 Hz, 8H), 4.73 (s, 2H), 7.05 (t, *J* = 1.7 Hz, 2H), 7.19 (d, *J* =7.8 Hz, 8H), 7.20 (d, *J* = 1.7 Hz, 4H), 7.31-7.34 (m, 6H), 7.40 (t, *J* = 7.6 Hz, 1H); ^13^C-NMR (125 MHz, CDCl_3_) δ 24.25 (q), 24.29 (q), 28.2 (t), 30.5 (t), 122.5 (d), 127.5 (d), 127.9 (d), 128.7 (d), 129.9 (d), 130.0 (d), 132.9 (s), 138.8 (s), 140.1 (s), 140.6 (s), 143.3 (s), 146.8 (s); ^77^Se-NMR (95 MHz, CDCl_3_) δ 419.7; UV-vis (CHCl_3_, 298 K) λ_max_ 511 nm (ε =153); LRMS (FD-TOF) *m/z* 1090 (M^+^). Anal. Calcd for C_67_H_79_ISe: C, 73.80; H, 7.30. Found: C, 73.82; H 7.23. For the ^1^H and ^13^C-NMR spectra, see Supplementary Materials.

### 3.2. Heating of ***2*** in [D_8_]toluene

A solution of **2** (5.5 mg, 5.0 µmol) in [D_8_]toluene (0.5 mL) in a 5 mm o/d NMR tube with a J-young valve was heated at 60 °C for 16 h. In an independent experiment, a solution of **2** (5.5 mg, 5.0 µmol) in [D_8_]toluene (0.5 mL) was heated at 100 °C for 3 h. In both cases, no change was observed in the ^1^H-NMR spectra.

### 3.3. Reduction of ***2*** with Dithiothreitol

To a solution of **2** (37.7 mg, 34.6 µmol) in CHCl_3_ (3 mL) in a Schlenk flask was added dithiothreitol (12.3 mg, 79.7 µmol) and triethylamine (11 µL, 0.11 mmol). The mixture was degassed through three freeze-pump-thaw cycles, and the flask was flushed with argon. The degassed solution was stirred at 40 °C for 15 h and then quenched with 5% aq. NH_4_Cl. After extraction with CHCl_3_, the combined organic layer was dried over MgSO_4_, and the solvent was evaporated to give the crude mixture. Purification by PTLC (hexane:CHCl_3_ = 4:1) afforded **1** (22.2 mg, 23.0 µmol, 66.7%) as colorless solids.

### 3.4. Hydrolysis of ***2***

A solution of **2** (29.5 mg, 27.1 µmol) in THF (1 mL) in a Schlenk flask was degassed through three freeze-pump-thaw cycles, and the flask was flushed with argon. To the solution was added an aqueous solution of NaOH (1.4 M, 117 µL, 0.16 mmol) at 0 °C, and the reaction mixture was stirred at 0 °C for 1 h. Quenching and work-up were carefully performed under an argon atmosphere. Thus, the resulting white suspension in an ice bath was treated with phosphate buffer (pH 7, 0.4 mL) followed by degassed water (0.8 mL). After extraction with degassed CHCl_3_ (1 mL × 3), the combined organic layer was dried over MgSO_4_. Evaporation of the solvent afforded selenenic acid **4** [13] as white solids (24.7 mg, 25.2 µmol, 93%). 

## 4. Conclusions

A primary-alkyl-substituted selenenyl iodide was successfully synthesized by taking advantage of a cavity-shaped steric protection group. Selenenyl iodides are usually unstable because of facile disproportionation to the corresponding diselenides and iodine. In contrast, the selenenyl iodide synthesized in this study showed remarkable thermal stability despite being a primary-alkyl derivative, demonstrating the effectiveness of the steric protection due to the cavity-shaped substituent. By utilizing the stable selenenyl iodide, a model reaction for a chemical process related to the function of an iodothyronine deiodinase was examined. Hydrolysis of the selenenyl iodide under alkaline conditions cleanly led to the formation of the corresponding selenenic acid, corroborating the chemical validity of the recent proposal that hydrolytic conversion of a selenenyl iodide to a selenenic acid is potentially involved in the catalytic mechanism of ID-3.

## Supplementary Materials

Supplementary materials can be accessed at: http://www.mdpi.com/1420-3049/20/12/19773/s1.
