# Supplementary material for: Synthesis of a Stable Primary-Alkyl-Substituted Selenenyl Iodide and Its Hydrolytic Conversion to the Corresponding Selenenic Acid
Source: Molecules. 2015 Dec 2;20(12):21415–20. doi: 10.3390/molecules201219773 (PMC6331843; doi:10.3390/molecules201219773)
Supplement: Supplementary file 1 [file molecules-20-19773-s001.pdf]

Supplementary Materials: Synthesis of a Stable Primary-Alkyl-Substituted Selenenyl Iodide and Its Hydrolytic Conversion to the Corresponding Selenenic Acid

Shohei Sase, Ryo Kakimoto, Ryutaro Kimura and Kei Goto

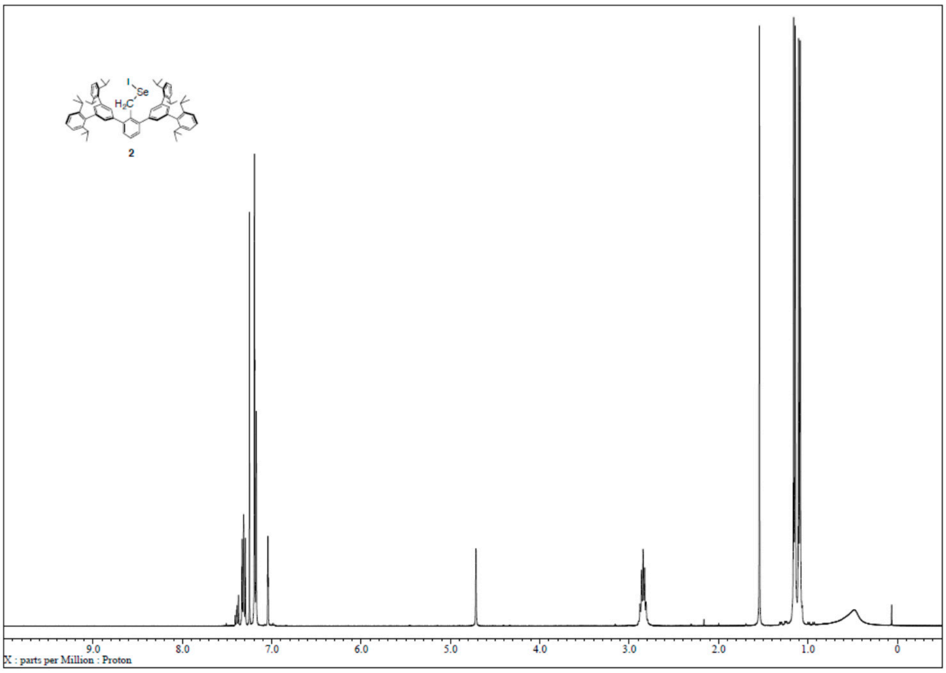

Figure S1. ^{1}H-NMR spectrum of compound 2 in $CDCl_3$.

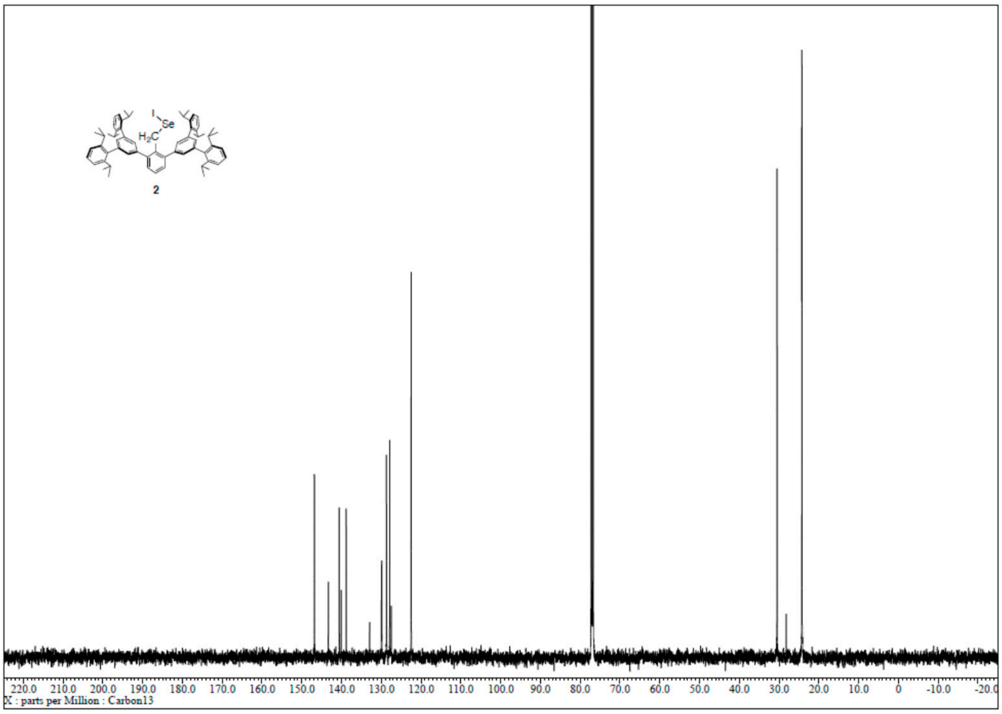

Figure S2. ^{13}C-NMR spectrum of compound 2 in $CDCl_3$.
